# Widely-Targeted Metabolic Profiling in *Lycium* *barbarum* Fruits under Salt-Alkaline Stress Uncovers Mechanism of Salinity Tolerance

**DOI:** 10.3390/molecules27051564

**Published:** 2022-02-26

**Authors:** Xiaojie Liang, Yajun Wang, Yuekun Li, Wei An, Xinru He, Yanzhen Chen, Zhigang Shi, Jun He, Ru Wan

**Affiliations:** 1 National Wolfberry Engineering Research Center, Yinchuan 750002, China; lxj910303@126.com (X.L.); liyuekun@nwberc.com.cn (Y.L.); anwei@nwberc.com.cn (W.A.); hexinru@nwberc.com.cn (X.H.); chenyanzhenok@163.com (Y.C.); hejun@nwberc.com.cn (J.H.); wanru@nwberc.com.cn (R.W.); 2 Ningxia Academy of Agriculture and Forestry Sciences, Yinchuan 750002, China; shizhigang@nwberc.com.cn

**Keywords:** metabolomics, nutritional quality, abiotic stress, goji berry

## Abstract

Wolfberry (*Lycium barbarum* L.) is an important economic crop widely grown in China. The effects of salt-alkaline stress on metabolites accumulation in the salt-tolerant *Ningqi1* wolfberry fruits were evaluated across 12 salt-alkaline stress gradients. The soil pH, Na^+^, K^+^, Ca^2+^, Mg^2+,^ and HCO_3_^−^ contents decreased at a gradient across the salt-alkaline stress gradients. Based on the widely-targeted metabolomics approach, we identified 457 diverse metabolites, 53% of which were affected by salt-alkaline stress. Remarkably, soil salt-alkaline stress enhanced metabolites accumulation in wolfberry fruits. Amino acids, alkaloids, organic acids, and polyphenols contents increased proportionally across the salt-alkaline stress gradients. In contrast, nucleic acids, lipids, hydroxycinnamoyl derivatives, organic acids and derivatives and vitamins were significantly reduced by high salt-alkaline stress. A total of 13 salt-responsive metabolites represent potential biomarkers for salt-alkaline stress tolerance in wolfberry. Specifically, we found that constant reductions of lipids and chlorogenic acids; up-regulation of abscisic acid and accumulation of polyamines are essential mechanisms for salt-alkaline stress tolerance in Ningqi1. Overall, we provide for the first time some extensive metabolic insights into salt-alkaline stress tolerance and key metabolite biomarkers which may be useful for improving wolfberry tolerance to salt-alkaline stress.

## 1. Introduction

Metabolites are minute biological molecules resident in cells acting as phenotypic markers in biological systems. Exceptionally valuable evidence from their metabolic pathways in diverse biological processes and functions in cell perturbations have previously been potentiated [1,2]. The diversity of metabolites in crops echoes not only the availability of their parallel genetic information, but also multi-substrate specificities for enzymes, non-enzymatic reactions, and subcellular compartmentation. The rising levels of environmental stresses have rendered metabolome time-dependent, and extremely subtle to copious ecological conditions [3,4]. There are two major groups of phyto-metabolites: primary metabolites which are indispensable for plant growth and development, and secondary metabolites which are crucial for plant survival under abiotic and/or biotic stresses and interactions with the external environment. The ability of higher plants to synthesize diverse metabolites with mixed biochemical complexity and physiological roles are integral in mitigating stress. Phyto-metabolites such as amino acids (proline), dimethylsulfonium compounds, glycine betaine, mannitol and sorbitol, and sugars (fructans, sucrose and trehalose) serve as osmolytes for osmoprotection under salinity stress [5,6]. When plants with increased concentrations of these osmolytes are subjected to salinity stress, they showed significant tolerance to stress [7,8,9]. Diversity of phyto-metabolites in wolfberry may provide novel defense compounds as biomarkers for deciphering their stress defense mechanisms. This has direct agronomic applications for developing potent biomarkers for breeding against stress. The field of metabolomics has direct applications in plant cell physiology and environmental research [10,11]; however, metabolite detection and stress-driven accumulation of metabolites in plants has received less research attention. Currently, metabolomics is gaining prominence in the study of biogenic volatile organic compounds (BVOCs) as specialized metabolites for biomarker development in specific plant species [12]. BVOCs are key signals in plant–plant interactions and are characteristically unleashed from roots into the soil and from flowers, fruits, leaves into the atmosphere, attracting pollinators and seed dispersers. Thus, plant metabolomics facilitates agrobiodiversity screening, a potential tool in studying plant stress defense mechanisms and biomarker development in breeding against abiotic stresses [13,14].

Neglected and underutilized species (NUS) are potential sources of useful economic traits and agronomically important genes for improving existing genotypes, particularly in the spate of looming climate variabilities. Wolfberry (*Lycium barbarum* L.), also referred to as Goji berry, is a Solanaceae tree species commonly farmed in the arid and semi-arid tropics of Africa, Eurasia, North and South America [13,14,15,16]. Seven species with three cultivated varieties are broadly distributed across the northwest and northern parts of China [15,17,18]. Approximately 101,171 ha of cultivated areas of elite genotypes were harvested in 2008 from China [15,17]. This increased in 2019 to 133, with 105 ha of cultivated areas under wolfberry husbandry across the country [13]. Wolfberry is prominent for its unique antioxidants, cosmetic, nutritional, pharmaceutics [13,19,20], minerals, metabolites and vitamin compositions [3,15,17,21,22]. In China, the fruit is put to assorted uses, including direct consumption either in the raw, cooked or dried forms [3,14,16]. The fruits are usually sun-dried for use in herbal teas, potages, hot pots, local juices, folk medicines and wines in the off-seasons [14,16]. *Lycium barbarum* polysaccharide (LBP), a wolfberry-specific antioxidant, is a highly sought-after phyto-based natural antioxidant in the fruits [23] with potential anticancer, anti-diabetic and anti-hyperglycemic activities [24,25,26,27]. Again, about 11 essential amino acids have been identified in the fruits [13,28,29,30], making it a powerhouse of nutrients for health. Several other polyphenols, such as alkaloids, cyclic peptides, flavonoids, lignanamides, lignans, terpenes and phenolic glycosides have been reported in the species [13,14,30].

Globally, agricultural lands are estimated to be lost to salt-alkalization at a rate of 0.25–0.5 Mha yearly [13]. The attendant environmental and socio-economic threats this poses is near alarming proportions. For instance, salt or alkali stress has been severally implicated in reduced photosynthetic efficiency [3]. The incessant accumulation of sodium bicarbonate (NaHCO_3_) precipitates alkalinity in soils. On the other hand, soil salinity is precipitated by buildup of sodium chloride (NaCl) in soils [13,31,32]. Soil alkalinity and salinity are commonly occasioned by evaporation, deforestation, overgrazing, and over-irrigation culminating in glaring secondary salinization, significantly impacting crop growth and productivity, particularly in Northwestern China [14,33,34,35]. The diverse agro-ecological climates across China gravely influence wolfberry cultivation and metabolites accumulation [12,14]. Even essential bioactive compounds such as total flavonoids, polyphenols and edible sugars are reportedly affected by varying climatic and micro environmental stress [7,36]. The impact of environmental stresses on constituents of crop species has recently gained notoriety in global climate change discourse [13]. Earlier studies in China focused on the agronomic performance of wolfberry (economic yield) under diverse agro-ecological climates [13,14]. It was against this backdrop that this study was undertaken to assess effects of salt-alkaline stress on metabolites accumulation in *Lycium barbarum* fruits using the widely-targeted metabolomics approach. We profiled several metabolites accumulated in ripe wolfberry fruits grown on typical salt-alkaline soil stress conditions and revealed key metabolic pathways engaged in response to the salt-alkaline stress. We provide for the first time some extensive metabolic insights into salt-alkaline stress tolerance and key metabolite biomarkers, which may be useful for improving wolfberry tolerance to salt-alkaline stress.

## 2. Results

### 2.1. Soil Physico–Chemical Properties along the Salt-Alkaline Stress Gradients

**Figure 1:**
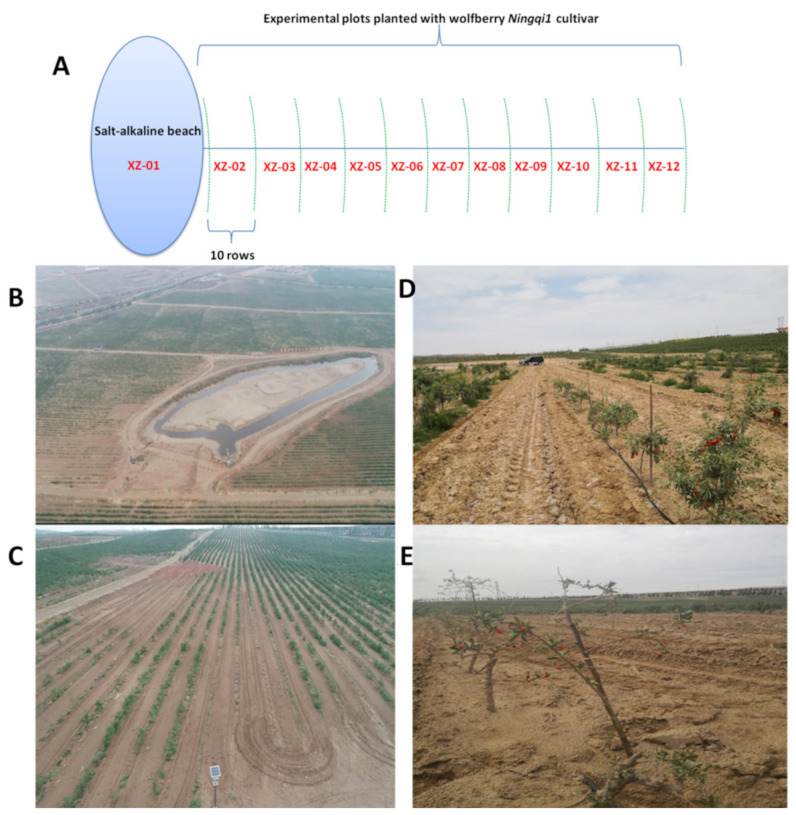
Field layout and experimental condition of the study area. (**A**) Experimental set up. Twelve salt-alkaline gradients were collected, numbered sequentially from XZ-01 to XZ-12. Each gradient consists of 10 rows of Ningqi1 trees; (**B**) Positions of the salt-alkaline beach in the field; (**C**) Definition of the stress gradients based on 10 parallel rows of wolfberry Ningqi1 trees away from the beach; (**D**,**E**) Wolfberry Ningqi1 trees under severe salt-alkaline stress.

A total of 36 soil samples along 12 salt-alkaline gradients were collected, numbered sequentially from XZ-01 to XZ-12 (Figure 1A). Taking the salt-alkaline beach as the center, the closer the surrounding wolfberry trees to the salt-alkaline beach, the more severe was the salt-alkaline stress (Figure 1). Wolfberry Ningqi1 trees grown in the XZ-01–XZ-05 gradients were all dead, while the trees grown in the XZ-06–XZ-12 gradients survived and produced fruits. The pH of soils from the study area spanned from 8.17 (XZ-12) to 9.03 (XZ-05) indicating the alkaline nature of these soils (Table 1). Higher soil alkalinity (pH) harms soil flora and fauna. Total soil salts (salinity) from the study areas ranged from 1.83 (XZ-12) to 33.90 (XZ-01). Essential cations (^+^) and anions (^−^) including potassium (K^+^), sodium (Na^+^), calcium (Ca^2+^), magnesium (Mg^2+^), chloride (Cl^−^), sulfate (SO_4_^2−^), and bicarbonate (HCO_3_^−^) ions were detected in soils of the study area. These ions varied significantly (*p* < 0.05) among the soils from the different gradients (Table 1). Soil samples from XZ-12 recorded the lowest Na^+^ (0.16 g/kg), while the highest Na^+^ (5.78 g/kg) was recorded in XZ-01. Soils from XZ-01 and XZ-02 registered the highest level of Ca^2+^ 2.34 g/kg and 2.33 g/kg, respectively, while XZ-11 and XZ-12 recorded the lowest levels of Ca^+^ 0.21 g/kg and 0.23 g/kg, respectively. The highest level of Mg^2+^ was recorded in soils from XZ-01 (2.11 g/kg); while the lowest Mg^2+^ levels were recorded in XZ-12 (0.08 g/kg), XZ-11 (0.09 g/kg), XZ-10 (0.18 g/kg), XZ-09 (0.18 g/kg). There was marked significant difference in Mg^2+^ accross the gradients. Soil porosity and permeability also has a key role in building up chlorides concentration. Excessive chloride concentration increase rates of corrosion of metals in irrigation system resulting in increased concentration of metals in soils [37]. Chloride concentration in soil samples from the study area varied between 0.23 g/kg (XZ-12) and 10.18 g/kg (XZ-01). Sulfate (SO_4_^2−^) occurs naturally in soils from water as a result of leaching from gypsum and other common minerals. The SO_4_^2−^ concentration in the soil samples varied between 0.61 g/kg (XZ-11) and 6.32 g/kg (XZ-01). Soils from XZ-01, XZ-02, XZ-03, XZ-04, XZ-07 showed highest HCO_3_^−^ concentrations and HCO_3_^−^ levels were statistically different across the stress gradients. These ionic species partially contributed to soil salinity and alkalinity.

### 2.2. Effects of Salt-Alkaline Stress on Wolfberry Fruit and Leaf Morphological Traits

Further analyses were conducted on samples from XZ-06–XZ-12 gradients as wolfberry trees grown in the XZ-01–XZ-05 gradients were all dead while trees grown in the XZ-06–XZ-12 gradients survived and produced fruits. Key fruit and leaf characteristics were employed for morphological assessment of the cultivars under salt-alkaline stress gradients. Globally, leaf length (cm), leaf width (cm), fruit weight (g), fruit longitudinal diameter (cm) and fruit transverse diameter (cm) decreased from the lowest stress gradients to the highest stress gradients, showing that salt-alkaline stress affects growth and yield of Ningqi1 (Table 2); however, it seems that the effect of salt-alkaline stress was not highly detrimental to Ningqi1 and it could bear soil salinity levels up to XZ-09 (Table 2).

### 2.3. Metabolic Profiles of Ripe Wolfberry Fruits from the Different Salt-Alkaline Gradients

**Figure 2:**
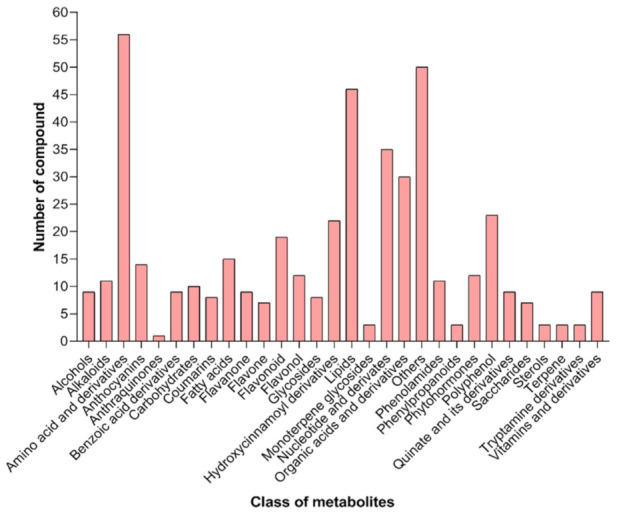
Metabolites detected in ripe wolfberry fruits based on metabolite compound clustering.

Overall, 457 metabolites were identified in the 21 ripe fruit samples collected from the six stress gradients (XZ-06–XZ-12) using the widely-targeted metabolomics technique (Figure 2). The metabolites were categorized into 29 recognized classes according to their biostructural conformations (Figure 2). Thus, the ripe wolfberry fruits were particularly rich in amino acids and derivatives (*n* = 56), lipids (*n* = 46), hydroxycinnamoyl derivatives (*n* = 22), nucleotides and derivates (*n* = 35), organic acids and derivatives (*n* = 30), polyphenols (including flavones, flavoves, flavonols, flavonoids, phenolamides and phenylpropanoids (*n* = 78), vitamins and derivatives, and several other classes of metabolites (*n* = 51; Figure 2).

**Figure 3:**
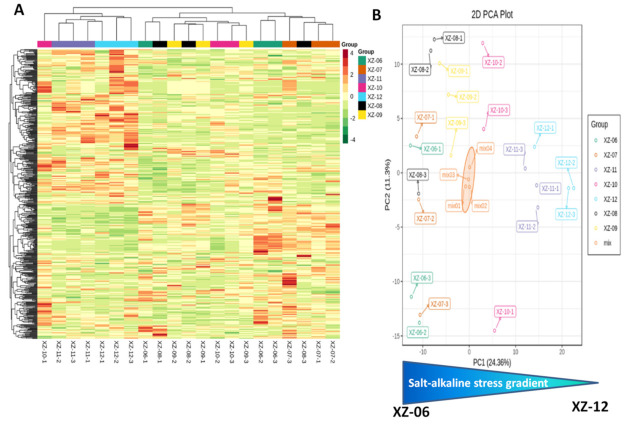
Profiles of metabolites in ripe fruits of wolfberry from 6 stress gradients. (**A**) Hierarchical clustering heatmap of metabolites based on ion intensity; and (**B**) principal component analysis (PCA). Six salt-alkaline gradients were collected, numbered sequentially from XZ-06 to XZ-12. Mix represents the mixed samples used for quality control. Log2 fold change of the metabolite ion intensity were used in the analyses. The salt-alkaline stress gradients were schematized below the PCA from the highest (XZ-06) to the lowest (XZ-12).

Ion intensities of metabolites were employed to heatmap the metabolites and a principal component analysis (PCA) was also performed on the samples (Figure 3). Most of the biological replicates from the same gradient were hierarchically clustered together, signifying the dependability of the metabolite profiling data (Figure 3A). In the PCA, the first two PCs accounted for approximately 36% of the total variability within the dataset (Figure 3B). PC1 distinguished samples from high salt gradients (XZ-06–XZ-09) and low salt gradients (XZ-10–XZ-12). Furthermore, the assorted samples used for quality control (mix) were all near origin (0:0), indicative of a very less technical variability within the dataset. 

### 2.4. Variation of Metabolome along Salt-Alkaline Gradients in Worlfberry Fruits

**Figure 4:**
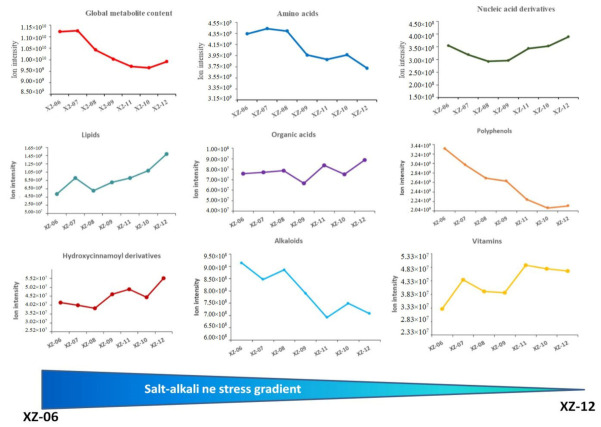
Major metabolite classes comparison between the different salt-alkaline stress gradients. These classes encompass approximately 80% of the total metabolites detected in the wolfberry fruits. Six salt-alkaline gradients were collected, numbered sequentially from XZ-01 06 to XZ-12. The salt-alkaline stress gradients were schematized below the line graphs from the highest (XZ-06) to the lowest (XZ-12).

We recorded a significant variation of metabolite ion intensity in fruits from the different stress gradients. Globally, the fruit metabolite concentration decreases as the soil salinity stress decreases, suggesting that soil salinity enhances metabolite accumulation in wolfberry (Figure 4). Amino acids and derivatives, alkaloids, and polyphenols (can be subdivided into six major groups, namely flavones, flavanones, flavonols, flavanols, phenolamides and phenylpropanoids) were more enriched in wolfberry fruits from XZ-06, XZ-07, XZ-08 and XZ-09. This suggests that these metabolites were induced by salinity stress, highlighting their probable role for salinity stress tolerance in Ningqi1. Conversely, we uncovered that nucleic acids, lipids, organic acids and derivatives, hydroxycinnamoyl derivatives and vitamins as significantly reduced by high salinity stress and therefore were highly concentrated in fruits from low salt stress gradients (XZ-10, XZ-11 and XZ-12) (Figure 4). 

### 2.5. Correlations between Soil Physico–Chemical Properties and Major Metabolite Classes 

Correlations between major metabolites and soil physico–chemical properties from the different stress gradients were analyzed (Table 3). Correlations between most of the metabolites and soil pH, salinity, Na^+^, Ca^2+^, Mg^2+^, Cl^−^ and SO_4_^2−^ were generally significant across the different salt-alkaline stress gradients. Soil pH, salinity, Na^+^, Ca^2+^, Mg^2+^, Cl^−^ and SO_4_^2−^ were positively correlated with amino acid derivatives, alkaloids, and polyphenols, evidencing direct roles of these metabolites in salinity tolerance in wolfberry. In contrast, soil physico–chemical properties negatively correlated with hydroxycinnamoyl derivatives, vitamins and lipids, suggesting their antogonistic influence in wolfberry tolerance to salinity stress. Overall, this result indicates that, wolfberry tolerance to salt-alkaline stress is influenced by soil physico–chemical properties and involves synergistic and/or antagonistic relationships with metabolites such as amino acids and derivatives, polyphenols, hydroxycinnamoyl derivatives, lipids, vitamins and alkaloids (Table 3).

### 2.6. Differential Metabolites Accumulation in Wolfberry Fruits from the Different Stress Gradients

**Figure 5:**
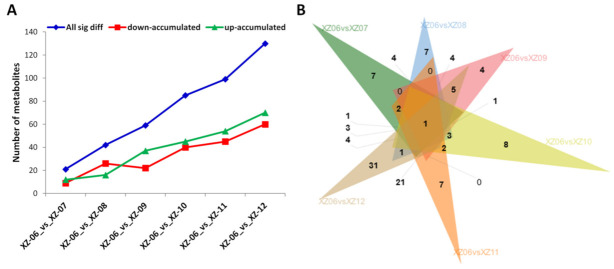
(**A**) Number of upregulated and downregulated differentially accumulated metabolites (DAM) between the different stress gradients. The blue line shows the difference in total DAMs content between the stress gradients. The green line represents the upregulated while the red line represents downregulated DAMs, respectively. (**B**) Venn diagram showing the number of shared and common DAMs between the different compared salt-alkaline stress gradients.

We analyzed wolfberry fruit metabolites that were differentially accumulated across the diverse salt-alkaline stress gradients under study. We retained as differentially accumulated metabolites (DAM) between two gradients, individual compounds with variable importance in projection (VIP) ≥1 and fold change (≥2 or ≤±0.5) (using partial least squares discriminant analysis (PLS-DA)) [38]. The salt-alkaline levels in the gradients substantially altered the metabolite content in the wolfberry fruits. We used the gradients with the highest soil salinity level (XZ-06) and compared with other gradients. From the analysis, 243 DAMs were recorded in total, ranging from 21 (XZ-06 vs. XZ-07) to 130 (XZ-06 vs. XZ-12) DAMs (Figure 5A), indicating that the higher the difference in soil salinity level between stress gradients, the higher the number of DAMs. Generally, approximately more than half of the global metabolome were substantially altered, indicating that the stress gradients as growth media impacted metabolites accumulation in the wolfberry fruits (Table 4). The number of upregulated DAMs was higher than downregulated DAMs at increasing levels of salt-alkaline stress gradients (Figure 5A), which confirms that high salinity stress induces metabolite accumulation in Ningqi1 fruits.

In total, 13 compounds (N-p-Coumaroylputrescine, Abscisic acid (ABA), LPC(1-acyl 18:2), dopamine hydrochloride, N′-*p*-Coumaroyl putrescine, lysoPC 16:2 (2n isomer), lysoPC 18:3 (2n isomer), (+)-cis,trans-Abscisic acid, cryptochlorogenic acid, lysoPC 15:1, 2,5-dihydroxy benzoic acid O-hexoside, chlorogenic acid (3-O-Caffeoylquinic acid), and lysoPC 16:1 (2n isomer)) were differentially accumulated between at least five out of the six compared stress gradients (Table 4), representing the core metabolites associated with salt stress responses in wolfberry, irrespective of the salinity stress levels. Remarkably, polyamines and ABA were increased with increasing salinity stress, implicating their positive functions in salinity tolerance (Table 4). Conversely, constant reductions of lipids and chlorogenic acids were observed with increasing salinity stress, suggesting that these metabolites are unfavorable for salt tolerance. Specifically, we identified LysoPC 16:1 (2n isomer) as the only metabolite persistently decreased in *Ningqi1* from the highest salt stress gradient to the lowest (Figure 5B). Overall, we propose these metabolites as potential biomarkers for salt stress tolerance in wolfberry.

## 3. Discussion

### 3.1. High Soil Salt-Alkaline Levels Improve Ningqi1 Wolfberry Fruit Quality

Saline and alkaline soils (6.7 × 10^6^ hm^2^) cover approximately 7% of cultivated lands in China [13]. Alkalization, salinization, and mixed salt-alkaline stresses in soils pose injurious effects to crop development in salinity-prone regions. The growth, development, and differentiation of plants on salt-alkaline soils are commonly restricted by salinity and pH levels, which is inimical to productivity of crops [14]. We analyzed 36 soil samples from 12 salt-alkaline stress gradients (sequentially from XZ-01 to XZ-12) for their physico–chemical characteristics. Wolfberry fruits harvested from XZ-06–XZ-12 gradients were profiled for their metabolites accumulation under the salt-alkaline soil stress conditions. Our results revealed that pH marginally varied from gradients XZ-01 to XZ-12 (Table 1) but decreased along the total salt concentration gradients. Salinity and pH have collinearity in alkaline soils; especially when carbonates are the main salt forms and within 9.5–10.5 pH ranges [39]. In this study, the salt predominantly consisted of chlorides and/or sulfates with some degree of relationship between salt content and pH. Thus, this finding strongly surmises that pH is equally an essential driver of salt-alkaline stress. The impacts of salt stress on physiological processes in plants and soil microbiota have been widely reported [2]. Total salt content recorded in this study varied significantly across the different stress gradients (Table 1). Moreover, variable concentrations of Ca^2+^, Mg^2+^, Cl^−^, SO_4_^2−^, and HCO_3_^−^ recorded from the different stress gradients in this study contributed diversely to the salinity and alkalinity stress. This is consistent with earlier studies that Ca^2+^, Cl^−^, HCO_3_^−^, Mg^2+^, and SO_4_^2−^ are key ions in saline or alkaline stress [40,41]. To reduce water loss under saline condition, plants can regulate leaf stomatal conductance to reduce water evaporation and increase photosynthetic efficiency. In this study, key morphological traits of leaf and even fruits did not significantly change under the salt-alkaline stress gradients (Table 2). This is consistent with findings by Yajun et al. [17] that, the wolfberry cultivar, *Ningqi1* is tolerant to salinity and alkaline conditions [42].

It is well known that abiotic stresses promote the biosynthesis of a wide range of bioactive compounds in plants, serving as functional molecules for crop adaptation, but also have a great interest for their beneficial effects on human health [21,43]. The fruits under high salt stress had more metabolites than fruits under low salt stress gradients, making it more nutritious (high accumulation of amino acids, alkaloids, organic acids, and polyphenols). Wolfberry fruits are reported to contain 18 amino acids, comprising all eight essential amino acids. The major amino acids in wolfberry fruit includes Ala, Asp, Glu, Gly, Lys, Pro, Ser, and Tyr involved in multiple functions in humans as antioxidants, DNA and RNA stabilization, growth factors, metabolic regulators, nutrients, and secondary messengers [44]. Alkaloids are vast naturally occurring metabolites containing nitrogen atoms in their structures. Alkaloids are implicated in alkalinity partly as a result of their nitrogen atoms and copiously associated with analgesics, anticancer, anti-inflammatory, antimicrobial, antifungal, anesthetic and pain relief, dietary ingredients and supplements, pharmaceuticals, and neuropharmacologic activities in humans [17,45]. Organic acids in wolfberries are responsible for taste and serve as an index of fruit ripeness which influences user tolerability [46]. Previous studies have reported that wolfberry predominantly contains fumaric, citric, malic, shikimic and tartaric acids as organic acid [14]. Polyphenols are organic compounds in wolfberry. Flavonoids, flavones, flavanones are common polyphenols detected in the species and are noted for their role in regulation of metabolism, and cell proliferation. Their antioxidant and anti-inflammatory properties make them useful in prevention of cancer, cardiovascular disease, neurodegenerative disorders, and obesity [30]. The accumulation of these metabolites under salt-alkaline stress improves the nutritional quality of *Ningqi1* wolfberry fruits. These results could promote wolfberry production on salt-alkaline lands as a way of marginal land valorization. Nonetheless, it is important to confirm our results on different wolfberry cultivars, especially salt-alkaline sensitive cultivars.

### 3.2. Wolfberry Metabolic Alteration in Response to Salinity Stress

The wolfberry cultivar, *Ningqi1* is a resilient genotype able to tolerate several abiotic stresses [13], including salt-alkaline stress; hence, studying its metabolic responses to salinity stress can provide significant and novel insights into its abiotic stress tolerance mechanisms in plants. The levels of numerous metabolites were altered under different salt-alkaline stress gradients, offering an excellent opportunity to identify novel salt-responsive compounds [12]. We detected that the salt-responsive metabolites in the wolfberry cultivar were both primary and secondary metabolites (amino acid and derivatives, hydroxycinnamoyl derivatives, nucleotide and derivates, organic acids, vitamins and derivatives, flavonoids, lipids and polyphenols). Primary metabolites are the most essential stress-affected metabolites under limited CO_2_ assimilation [12]. Consequently, salt stress has been repeatedly reported to significantly impact primary metabolism in barley [35], maize [47], rice [48] and peanut [49]. Secondary metabolites, including flavonoids, lipids, polyphenols detected in this study play a crucial role in biotic and abiotic stress responses in the wolfberry cultivar. Several studies have revealed that the synthesis of flavonoids, lipids, and polyphenols (secondary metabolites) is greatly swayed by salinity stress [9]. Thus, these secondary metabolites could serve as the non-enzymatic scavengers against reactive oxygen species (ROS) to shield cellular structures and macromolecules in wolfberry. 

Plants accumulate compatible solutes (low-molecular-weight metabolites) such as amino acids, carbohydrates and polyphenols, and organic acids to adapt to osmotic balance between their cytoplasm and environment. Amino acids are used by plants to ameliorate osmotic adjustment and maintain cell membrane stability. Amino acids play prominent functions in protein biosynthesis, and represent the building blocks for several biosynthetic pathways and participate in signaling processes during plant stress response [14]. The nucleotide metabolism is key to plant life as building blocks of nucleic acids synthesis, energy sources, co-enzymes for redox reactions and precursors for synthesis of primary and secondary products [12]. Moreover, organic acid metabolism is central for various biochemical pathways, including energy production, formation of precursors for amino acid biosynthesis and cellularly modulating adaptation of plants to abiotic stresses [30,44]. Organic acid metabolism also acts as photosynthetic intermediates in plants and as metabolically active solutes for osmotic adjustment and balance of cation excess. Thus, their activity is crucial for plant responses to salinity stress. Under the salt-alkaline stress, amino acid metabolism was greatly enriched, revealing that amino acid metabolism in the *Ningqi1* fruits likely contributed to enhanced salinity tolerance. In addition, aminoacyl tRNA biosynthesis, flavonoid biosynthesis, glycerophospholipids metabolism, phenylpropanoid biosynthesis and proline synthesis pathways had the same trends as amino acid metabolism, indicating that *Ningqi1* fruits could synthesize more nitrogen-containing compounds to cope with salinity stress [12]. The wolfberry cultivar *Ningqi1* also reduced the TCA cycle, organic acids metabolism to save energy in order to counter salt-alkaline stress [12]. 

### 3.3. Key Salinity-Tolerant Biomarkers Identified in Wolfberry

**Figure 6:**
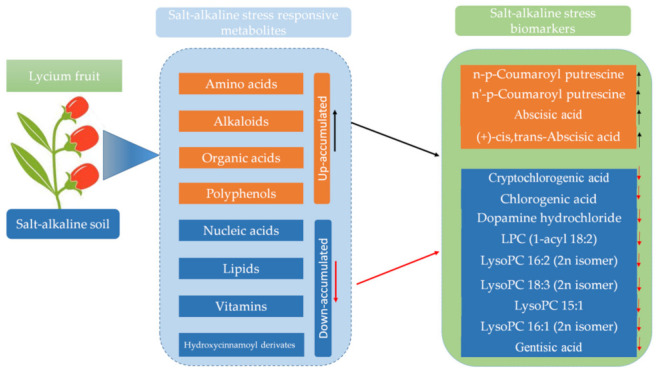
Summary of the metabolic responses to salt-alkaline stress in Lycium barbarum fruit. Under salt-alkaline stress, several classes of metabolites are upregulated or downregulated: salt-alkaline responsive metabolites. Thirteen metabolites are constitutively differentially accumulated across various salt-alkaline stress gradients: salt-alkaline stress biomarkers. The black arrow stands for upregulated metabolites while the red arrow stands for downregulated metabolites.

Candidate metabolites as stress-tolerant biomarkers are treasured tools in crop improvement programmes [12]. Overall, we identified 13 metabolites as key salinity-tolerant biomarkers in *Ningqi1* which could be further validated in a large sample of wolfberry cultivars (Figure 6). These metabolites were classified as lipids, phytohormone, quinate and derivatives and polyamine; however, all of them did not follow the same trend of accumulation across the salt-alkaline stress gradients, suggesting a complex mechanism for salt tolerance in *Ningqi1*. 

Abscisic acid (ABA) was constitutively upregulated across the stress gradients. ABA is a key phytohormone reputed for enhancing plant adaptation to salinity stress [48]. High salinity increases ABA biosynthesis [50]. Calcium dependent protein kinases (CDPK), mitogen activated protein kinase (MAPK) cascades, receptor-like kinases (RLK), sucrose non-fermenting-1 (SNF1)-related protein kinases, transcription factors and microRNAs (miRNAs) have been established as key ABA-dependent pathways under salinity stress [48]. ABA up-regulation stimulates dependent pathways as essential salt sensory pathways in the tolerant *Ningqi1* cultivar to regulate expression of various genes that may eventually influence its salinity tolerance levels [48,50]. Thus, ABA signaling pathways can therefore be considered essential to salt stress response in wolfberry.

Polyamine and phenolamide (n-p-coumaroyl putrescine and n′-p-coumaroyl putrescine) were detected as important positive biomarkers for salinity tolerance in wolfberry. Phenolamide and polyamines have long been implicated in range of biological processes including plant development and defense against dehydration, salinity, mineral deficiency stresses, and ultra-violet radiations [35]. Putrescine is a precursor to spermidine, and spermine (polyamines) and is significant for its role in plant stress responses. Putrescine is synthesized from arginine via either arginine decarboxylase or orthinine decarboxylase. Putrescine decreased significantly in response to salinity stress in the elongation zone in clipper and Sahara [35]. Contradicting results have been reported for putrescine accumulation in response to salt stress in rice. Many studies have established increased putrescine accumulation in roots of tolerant rice varieties; while others have reported decreased accretion in sensitive rice varieties [35]. Widodo et al. [51] reported an increase in putrescine after 5 weeks of salt stress in barley leaves associated with leaf senescence and cell damage. Putrescine reportedly decreased in roots but increased in the leaves in mung bean in response to salt stress [52]. These varying results of putrescine accumulation in response to salt stress may be attributed to tissue sampling procedures and thus, necessitating spatial metabolomics analyses. 

Five lipids or fatty acids (LPC(1-acyl 18:2), LysoPC 16:2 (2n isomer), LysoPC 18:3 (2n isomer), LysoPC 15:1, and LysoPC 16:1 (2n isomer)) were significantly downregulated with increasing salt-alkaline stress in *Ningqi1*. Lipids or fatty acids are the main components of cell membrane that enhance cell fluidity in response to stress. In fact, fatty acids represent the sole component of membrane lipids, regulating membrane fluidity and vital in salt tolerance of plants [53]. Changes in the membrane lipid or fatty acid composition would alter membrane permeability, membrane potential, and activities of membrane-bound enzymes. Furthermore, lipids or fatty acids are also involved in scavenging excess production of reactive oxygen species (ROS) as these extremely reactive molecules obstruct the redox system and activate prime metabolic pathways by directly altering enzymatic activity, DNA structure and membrane properties [53]. Earlier studies evidence that lipids serve as a mobile rescue station for cytoplasmic components recycling, membrane reconstruction and protein storage in response to salinity stress [53]. Thus, the downregulation of lipids in the fruits might be crucial as a protective mechanism for the survival of wolfberry cells under salt stress and also for a continued cellular activity of the cultivar as lipid level is an indicator of cell state. 

Chlorogenic acids synthesis is a cost-effective strategy for alleviating growth reduction and salinity-induced oxidative damage in halophytes. Accumulation of chlorogenic acids in leaves of honeysuckle was established as an important tolerance mechanism in response to salinity stress [54]. The down-accumulation of chlorogenic acid in the fruits may be a mechanism against salinity-induced growth reduction in the wolfberry cultivar. Further analysis is required to understand how downregulation of chlorogenic acids contributes to salt-alkaline stress tolerance in wolfberry. Finally, accumulation of 2,5-dihydroxy benzoic acid o-hexoside (gentisic acid) was significantly repressed with increasing salt-alkaline stress levels. Gentisic acid is reported to be a pathogen-induced signal molecule for plant defense response [55]. In rice, gentisic acid could not be detected in the tolerant varieties without salt stress, but was induced after NaCl treatment in salt-tolerant varieties [55]. Therefore, the metabolite may be acting as a signaling molecule in response to salinity stress in the tolerant wolfberry cultivar. De novo production of gentisic acid (secondary metabolite) in natural halophytes such as wolfberry (salt-tolerant species) presents it as a potential salt-tolerant biomarker [56]; however, further study is required to unravel the specific role of gentisic acid in salinity tolerance in wolfberry.

## 4. Materials and Methods

### 4.1. Plant Materials, Study Site and Sampling 

The salt-tolerant cultivar *Ningqi1*, commonly cultivated in Northwest China, was used as experimental material [1,27]. The study was led during summer on the commercial farms of wolfberry Company Ltd. in Xiaoyanchitan Xizan Manor, Hongliugou production zone, Mingsha Town, Zhongning County, Ningxia Hui autonomous region of China with longitude 40°09′00′′ N, and latitude 94°40′00′′ E (Figure 1). Twelve soil salt-alkaline gradients were sampled by taking the beach as the center: XZ-01 was from a salt-alkaline beach bottom, which was outside the experimental plot (Figure 1). XZ-02, XZ-03, XZ-04, XZ-05, XZ-06, XZ-07, XZ-08, XZ-09, XZ-10, XZ-11 and XZ-12 were the positions of the 1st, 11th, 21st, 31st, 41st, 51st, 61st, 71st, 81st, 91st and 101st rows of trees, respectively, in the wolfberry field away from the beach. Soil samples (0–40 cm of the root distribution) were collected at four locations in each gradient. In contrast to XZ-01–XZ-05, trees from XZ-06–XZ-12 gradients survived and produced fruits. The leaves and fruits were therefore collected on wolfberry trees from XZ-06–XZ-12 stress gradients and compared.

### 4.2. Soil Physico–Chemical Analyses

A total of 36 soil samples along the 12 gradients were collected and the physico–chemical properties were determined at the Ningxia Zhilian Institute of Detection Science and Technology, China. Among them, the soil pH test was done based on LY/T 1239-1999 method [57]; the total soil salts were estimated using LY/T 1251-1999 method [58]; potassium and sodium ions were determined by flame spectrophotometry (FSP) [59]; calcium and magnesium ions were determined by the atomic absorption spectrometry method (AAS) [60]; chloride ions were determined using the silver nitrate titration method (SnT) [61]; sulfate ions were detected using the barium sulfate turbidimetric method (BSTD) [62]; carbonate and bicarbonate ions were determined using the dual indicator titration method (DIT) [63]. 

### 4.3. Fruit and Leaf Survey

The collection of fruits was done simultaneously with the collection of soil samples. Centrifugal tubes were used to collect three disease-free mature ripe fruits from the wolfberry trees at different positions on each gradient (XZ-06–XZ-12). Labeled samples were kept in liquid nitrogen and transported to the laboratory and stored in a −80 °C refrigerator for metabolome analyses. Under the same treatment, fruits and leaves were picked from different individuals and then divided into three samples. The single fruit weight, longitudinal and transverse diameters were measured on 15 random fresh fruits. In addition, the length and leaf width of 15 random leaves was measured. The surveys were conducted in July 2019.

### 4.4. Fruit Metabolome Analyses

Widely-targeted metabolome analyses were performed by the Wuhan MetWare Biotechnology Co., Ltd. (Wuhan, China) on the wolfberry fruits harvested from the different salt-alkaline stress gradients. Each gradient sample consisted of 3 biological replicates, with a total of 21 samples.

#### 4.4.1. Metabolome Profiling

Sample preparation and analysis, metabolite detection and computations were undertaken by the Wuhan MetWare Biotechnology Co., Ltd. (Wuhan, China), following their standard procedures as described hitherto by Liu et al. [10].

#### 4.4.2. Sample Preparation for Metabolites Extraction 

Lyophilized fruit samples were pulverized using a mixer mill (MM 400, Retsch, Shanghai, China) with zirconia beads for 1.5 min at 30 Hz. The powdered samples (100 mg) powder was weighted and extracted with 1.0 mL 70% aqueous methanol. The sample was then stored at 4 °C overnight. During this time, the sample was vortexed six times to increase the extraction rate. Following centrifugation at 10,000× *g* for 10 min, the extracts were absorbed (CNWBOND Carbon-GCB SPE Cartridge, 250 mg, 3 mL; ANPEL, Shanghai, China) and filtrated (SCAA-104, 0.22 μm pore size; ANPEL, Shanghai, China) before LC–MS analysis.

#### 4.4.3. Metabolites Determination and Assessment

Sample extracts were evaluated using an LC-ESI-MS/MS system (HPLC, Shim-pack UFLC SHIMADZU CBM30A system, http://www.shimadzu.com.cn/, (accessed on 18 November 2019) Kyoto, Japan; MS, Applied Biosystems 4500 Q TRAP, http://www.appliedbiosystems.com.cn/, (accessed on 18 November 2019) San Diego, CA, USA). The analytical conditions were as follows, HPLC: column, Waters ACQUITYUPLC HSS T3 C18 1.8 μm, 2.1 mm × 100 mm; mobile phase: The aqueous phase is ultrapure water (adding 0.04% acetic acid), and the organic phase is acetonitrile (adding 0.04% acetic acid); gradient program, 95:5 *v*/*v* at 0 min, 5:95 *v*/*v* at 11.0 min, 5:95 *v*/*v* at 12.0 min, 95:5 *v*/*v* at 12.1 min, 95:5 *v*/*v* at 15.0 min; flow rate, 0.4 mL·min^−^^1^; temperature, 40 °C; injection volume: 5 μL. The HPLC effluent was alternatively connected to an electrospray ionization (ESI)-triple quadrupole-linear ion trap–MS/MS system (Applied Biosystems 4500 Q TRAP, San Diego, CA, USA). The ESI source operation parameters were as follows: ion source, turbo spray; source temperature 550 °C; ion spray voltage (IS) 5500 V; curtain gas (CUR) were set at 25.0 psi; the collision gas (CAD) was high. In triple quadrupole (QQQ), each ion pair is scanned according to the declustering potential (DP) and collision energy (CE).The analytical conditions were adapted from Chen et al. [64,65,66,67]. Metabolite quantification was done using multiple-reaction monitoring (MRM) [65] and the MetWare MWDB database based on their standard metabolic operating procedures [66].

### 4.5. Statistical Analyses

Quality control (QC) analysis was conducted to confirm the reliability of the data prior to the overall analyses. The QC sample was prepared by mixing sample extracts for insertion into every three samples to monitor the changes in repeated analyses. Data matrices with the intensity of the metabolite features from the 21 samples were uploaded to the Analyst 1.6.1 software (AB SCIEX, Ontario, Canada) for peak integration, and R software (version 3.3.2; www.r-project.org (accessed on 14 May 2020)) for statistical analyses. The partial least squares discriminant analysis (PLS-DA) was performed to maximize the metabolome differences between sample pairs, and R software (version 1.0.1; www.r-project.org (accessed on 14 May 2020) for statistical analyses. The relative importance of each metabolite to the PLS-DA model was tested using the variable importance in projection (VIP) as a parameter. Metabolites with VIP ≥ 1 and fold change ≥ 2 or fold change ≤ ±0.5 were considered as differential metabolites for group discrimination [67]. Pearson correlation analysis, principal component analysis (PCA, Table 3) and Ward’s hierarchical clustering heatmap were performed using R software (version 3.3.2; www.r-project.org (accessed on 14 May 2020) [68]. Consequently, a metabolic pathway was constructed according to KEGG (http://www.genome.jp/kegg/ (accessed on 28 April 2020) [69]; and pathway analysis was performed using MetaboAnalyst (http://www.metaboanalyst.ca/ (accessed on 28 April 2020) based on the change in metabolite concentration compared with the corresponding controls [67].

## 5. Conclusions

1. The soil pH, Na^+^, K^+^, Ca^2+^, Mg^2+^ and HCO_3_^−^ contents decreased proportionally across the salt-alkaline stress gradients. Based on the widely-targeted metabolomics approach, we identified 457 diverse metabolites, 53% of which were affected by salt-alkaline stress.
2. Soil salt-alkaline stress enhanced metabolites accumulation in wolfberry fruits. Amino acids, alkaloids, organic acids, and polyphenols contents increased proportionally across the salt-alkaline stress gradients. In contrast, nucleic acids, lipids, hydroxycinnamoyl derivatives, organic acids and derivatives and vitamins were significantly reduced by high salt-alkaline stress. A total of 13 salt-responsive metabolites represent potential biomarkers for salt-alkaline stress tolerance in wolfberry.
3. Specifically, we found that constant reductions of lipids and chlorogenic acids; up-regulation of abscisic acid and accumulation of polyamines are essential mechanisms for salt-alkaline stress tolerance in Ningqi1. Overall, we provide for the first time some extensive metabolic insights into salt-alkaline stress tolerance and key metabolite biomarkers, which may be useful for improving wolfberry tolerance to salt-alkaline stress.

## Figures and Tables

**Table 1:** Soil physico–chemical properties of the study area.

Stress Gradients	Soil Physico–Chemical Properties								
	pH	Salt	K^+^	Na^+^	Ca^2+^	Mg^2+^	Cl^−^	SO_4_^2−^	HCO_3_^−^
XZ-01	8.70 ^abcd^	33.90 ^a^	0.05 ^a^	5.78 ^a^	2.34 ^a^	2.11 ^a^	10.18 ^a^	6.32 ^a^	0.17 ^a^
XZ-02	8.87 ^ab^	17.77 ^b^	0.03 ^b^	2.74 ^bc^	2.33 ^a^	1.03 ^b^	4.58 ^b^	5.07 ^b^	0.15 ^b^
XZ-03	8.97 ^a^	12.83 ^c^	0.02 ^bc^	3.13 ^b^	0.61 ^cd^	0.86 ^bc^	4.22 ^b^	1.59 ^ef^	0.15 ^b^
XZ-04	8.93 ^ab^	13.33 ^c^	0.02 ^bc^	3.31 ^b^	0.56 ^cd^	0.90 ^bc^	4.54 ^b^	1.53 ^ef^	0.16 ^ab^
XZ-05	9.03 ^a^	8.93 ^de^	0.03 ^d^	2.10 ^cd^	0.64 ^cd^	0.51 ^d^	2.57 ^c^	1.53 ^ef^	0.12 ^cd^
XZ-06	8.77 ^abcd^	10.53 ^cd^	0.04 ^b^	1.71 ^de^	1.95 ^ab^	0.54 ^d^	1.99 ^cd^	3.59 ^c^	0.13 ^c^
XZ-07	8.90 ^ab^	10.87 ^cd^	0.05 ^a^	1.10 ^de^	1.64 ^b^	0.59 ^cd^	2.31 ^c^	3.34 ^cd^	0.16 ^ab^
XZ-08	8.83 ^abc^	6.57 ^ef^	0.06 ^a^	1.24 ^ef^	1.04 ^c^	0.36 ^de^	1.36 ^cde^	2.30 ^de^	0.13 ^c^
XZ-09	8.33 ^de^	5.30 ^efg^	0.05 ^a^	0.94 ^fg^	0.74 ^cd^	0.18 ^e^	1.02 ^de^	1.50 ^ef^	0.11 ^e^
XZ-10	8.47 ^bcde^	4.00 ^fg^	0.06 ^a^	0.58 ^fgh^	0.67 ^cd^	0.18 ^e^	0.47 ^e^	1.38 ^ef^	0.10 ^ef^
XZ-11	8.37 ^cde^	1.93 ^g^	0.05 ^a^	0.24 ^gh^	0.21 ^d^	0.09 ^e^	0.28 ^e^	0.61 ^f^	0.14 ^bc^
XZ-12	8.17 ^e^	1.83 ^g^	0.05 ^a^	0.16 ^h^	0.23 ^d^	0.08 ^e^	0.23 ^e^	0.66 ^f^	0.15 ^b^

XZ-01–XZ-12 refer to the various stress gradients. A column of data under one indicator with different lowercase letter are significantly different at *p* < 0.05 according to S-N-K ANOVA.Salinity, K^+^, Na^+^, Ca^2+^, Mg^2+^, Cl^−^, SO_4_^2−^ and HCO_3_^−^ were measured in g/kg.

**Table 2:** Effect of various salinity gradients on key wolfberry leaf and fruit traits.

Salt Stress Gradients	Leaf Traits		Fruit Traits		
	Length (cm)	Width (cm)	Weight (g)	Longitudinal Diameter (cm)	Transverse Diameter (cm)
XZ-06	4.03 ^a^	0.95 ^a^	0.49 ^a^	1.25 ^a^	0.92 ^a^
XZ-07	4.26 ^a^	1.05 ^a^	0.48 ^a^	1.43 ^a^	0.92 ^a^
XZ-08	4.89 ^ab^	1.13 ^ab^	0.36 ^a^	1.16 ^a^	0.83 ^a^
**XZ-09**	**5.16 ^b^**	**1.35 ^b^**	**0.74 ^b^**	**1.68 ^b^**	**1.08 ^b^**
XZ-10	4.37 ^a^	1.22 ^b^	0.83 ^b^	1.61 ^b^	1.00 ^b^
XZ-11	5.07 ^b^	1.33 ^b^	0.79 ^b^	1.51 ^b^	0.94 ^ab^
XZ-12	5.14 ^b^	1.28 ^b^	0.86 ^b^	1.70 ^b^	1.02 ^b^

Length = mean length; Width = mean width; weight = mean weight; Longitudinal diameter = mean longitudinal diameter; Transverse diameter = mean transverse diameter; XZ-01–XZ-012 refer to the various salt stress gradients of the wolfberry Ningqi1 trees. A column of data under one indicator with different lowercase letter are significantly different at *p* < 0.05 according to S-N-K ANOVA. The bolded values represent threshold of salinity tolerance.

**Table 3:** Correlations between soil physico–chemical properties and major metabolites in wolfberry fruits.

	Amino Acid Derivatives	Polyphenols	Hydroxycinnamoyl Derivatives	Lipids	Nucleic Acid Derivatives	Organic Acids	Vitamins	Alkaloids
pH	0.97 ***	0.78 **	−0.89 **	−0.73 **	−0.49	−0.18	−0.49	0.82 **
Salt	0.88 **	0.96 ***	−0.72 **	−0.70 **	−0.39	−0.32	−0.70 **	0.83 **
K^+^	−0.14	−0.49	0.04	0.07	−0.37	0.30	0.53 *	−0.37
Na^+^	0.83 **	0.96 ***	−0.75 **	−0.90 ***	−0.49	−0.42	−0.89 **	0.91 ***
Ca^2+^	0.85 **	0.97 ***	−0.68 *	−0.73 **	−0.30	−0.27	−0.73**	0.83 **
Mg^2+^	0.91 ***	0.92 ***	−0.75 **	−0.65 *	−0.33	−0.20	−0.62 *	0.83 **
Cl^−^	0.91 ***	0.94 ***	−0.76 **	−0.67 *	−0.44	−0.35	−0.68 *	0.85 **
SO_4_^2−^	0.89 **	0.96 ***	−0.72 **	−0.72 **	−0.34	−0.25	−0.71 **	0.85 **
HCO_3_^−^	0.29	0.11	−0.13	0.38	0.30	0.25	0.07	0.23

Values in bold refer to either * significant (*p* ˂ 0.05); ** very significant (*p* ˂ 0.001); and/or *** highly significant (*p* ˂ 0.0001) computed using Standard Linear Pearson Correlation.

**Table 4:** Consistently and differentially accumulated metabolites between wolfberry fruits from six salt-alkaline stress gradients.

Metabolite Class	Compound	Log2 Fold Change					
		XZ-06 vs. XZ-07	XZ-06 vs. XZ-08	XZ-06 vs. XZ-09	XZ-06 vs. XZ-10	XZ-06 vs. XZ-11	XZ-06 vs. XZ-12
Polyamine	N-p-Coumaroyl putrescine	−0.772	−1.081	−1.237	−1.272	−1.478	−1.987
Phenolamides	N′-p-Coumaroyl putrescine	−0.58	−1.288	−1.342	−1.449	−1.838	−2.444
Phytohormone	Abscisic acid	−0.991	−2.348	−2.376	−2.014	−2.304	−2.982
Phytohormones	(+)-cis,trans-Abscisic acid	−0.931	−2.472	−2.296	−1.916	−2.469	−2.743
Quinate and derivatives	Cryptochlorogenic acid	−0.269	1.8	2.209	1.539	2.253	3.678
Quinate and derivatives	Chlorogenic acid (3-O-Caffeoylquinic acid)	−3.187	2.006	2.641	1.668	2.445	3.922
-	Dopamine hydrochloride	−0.654	1.064	2.194	1.489	2.142	3.902
Fatty acid	LPC(1-acyl 18:2)	2.504	1.38	2.194	3.116	2.655	3.987
Lipids_glycerophospholipids	LysoPC 16:2 (2n isomer)	2.104	1.392	1.768	2.378	1.889	3.921
Lipids_glycerophospholipids	LysoPC 18:3 (2n isomer)	1.86	1.015	1.551	2.12	1.904	3.077
Lipids_glycerophospholipids	LysoPC 15:1	2.096	1.023	1.768	1.822	1.878	3.604
Lipids_glycerophospholipids	LysoPC 16:1 (2n isomer)	1.987	1.052	1.618	2.413	2.445	2.811
Benzoic acid derivatives	2,5-dihydroxy benzoic acid O-hexoside (Gentisic acid)	1.187	0.575	1.238	1.454	2.477	2.824

## Data Availability

Not applicable.
